# Supplementary figures and images for: Factors associated with poor outcomes among people living with HIV started on anti-retroviral therapy before and after implementation of “test and treat” program in Coastal Kenya
Source: PLoS One. 2022 Sep 21;17(9):e0270653. doi: 10.1371/journal.pone.0270653 (PMC9491584; doi:10.1371/journal.pone.0270653)

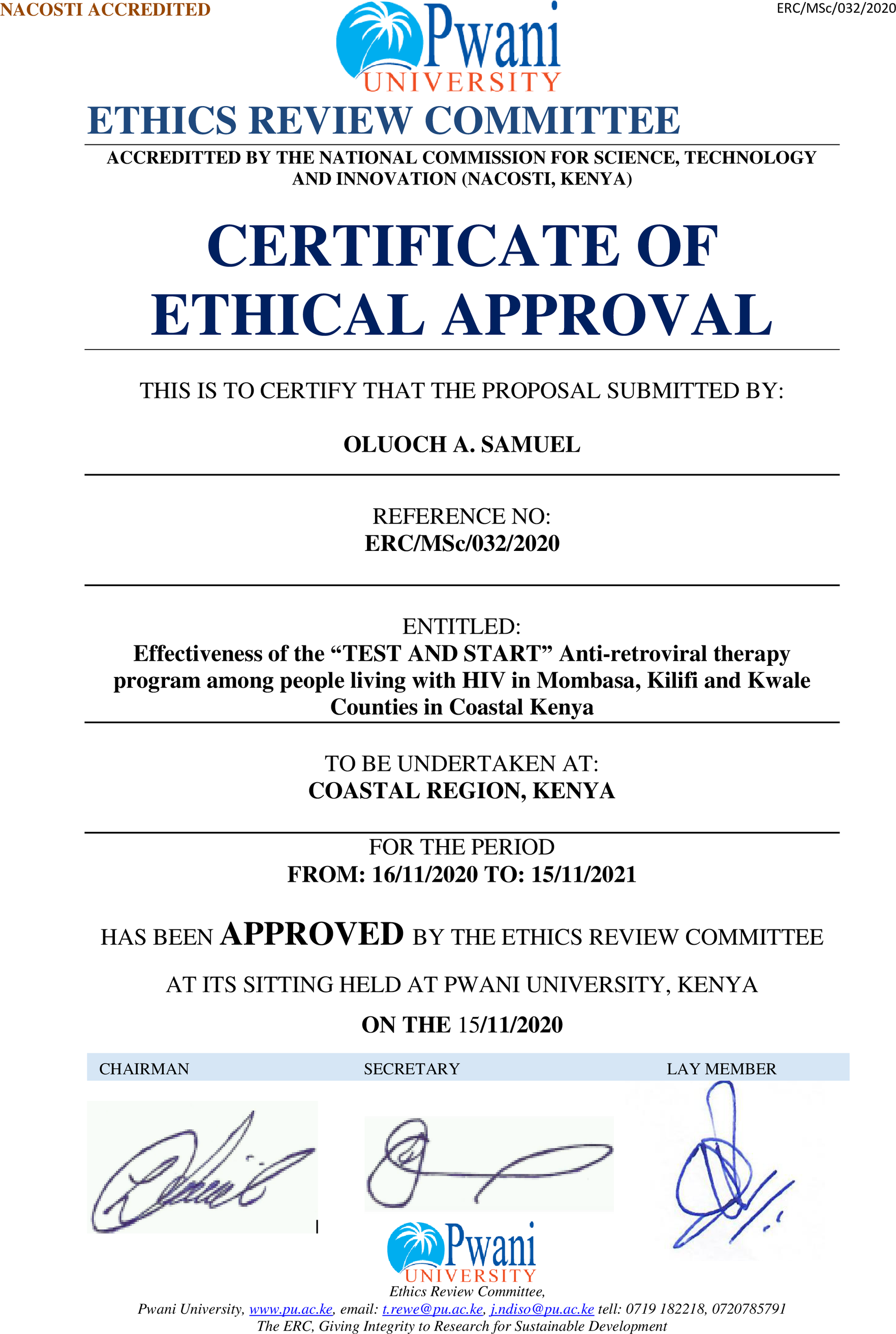

Supplement: S1 Ethical clearance — (TIF) [file pone.0270653.s001.tif]

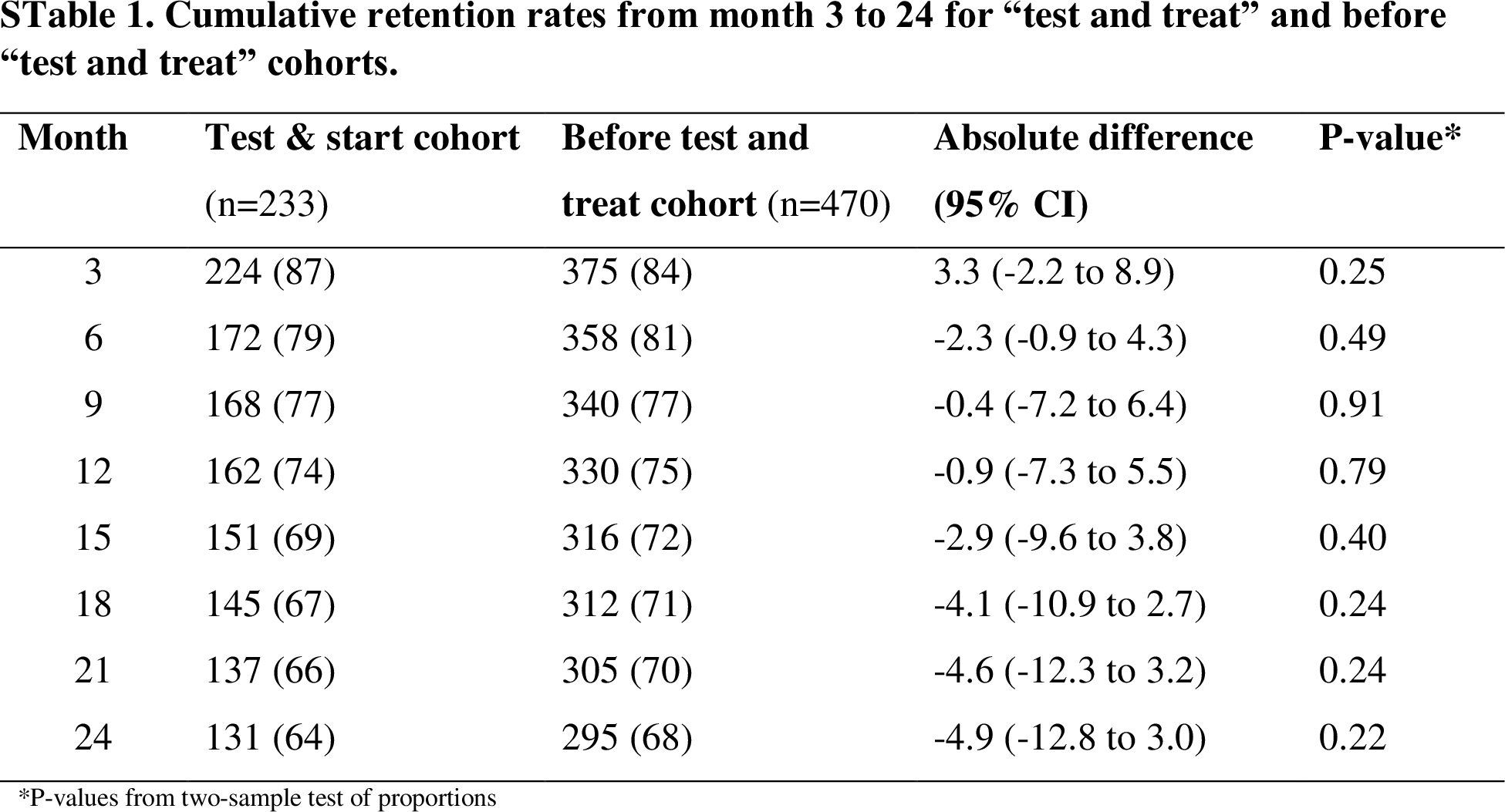

Supplement: S1 Table — (TIF) [file pone.0270653.s003.tif]
